# Face masks in action: Birds show reduced fear responses to people wearing face masks during the COVID-19 pandemic in three Asian countries

**DOI:** 10.1016/j.heliyon.2024.e24970

**Published:** 2024-01-23

**Authors:** Shuang Yang, Jianping Liu, Asif Sadam, Mominul Islam Nahid, Rahmat Ullah Khan, Wei Liang

**Affiliations:** a Ministry of Education Key Laboratory for Ecology of Tropical Islands, Key Laboratory of Tropical Animal and Plant Ecology of Hainan Province, College of Life Sciences, Hainan Normal University, Haikou, 571158, China; b College of Biological Sciences and Engineering, North Minzu University, Yinchuan, 750021, China

**Keywords:** Antipredator behaviour, COVID-19 lockdown, Face mask, Flight initiation distance, Habituation

## Abstract

The 2019 zoonotic pandemic (COVID-19), has led to a massive global lockdown that provides a good opportunity to study how wildlife responds to changes in human activity. Wearing a mask after the COVID-19 outbreak was widely used to prevent the spread of the causative pathogen. It has been shown that tree sparrows (*Passer montanus*) at two sites in south China exhibit reduced fear responses to people with face masks after a period of heavy exposure to them, whereas European studies showed the opposite, with no changes in the behaviour of the birds towards mask wearers in either rural or urban areas. To further study this, from October 2021 to January 2022, we conducted a flight initiation distance (FID) survey in Pakistan, Bangladesh, and Xi'an, China for a variety of field bird species by comparing the FID for researchers wearing masks to that for researchers not wearing masks to assess whether wearing masks in public places caused birds to adjust their flight response. Results from the three Asian countries showed that after a period of sustained contact with people wearing masks, in both rural and urban areas, birds were significantly more adapted to them and had a shorter FID to people wearing masks. We suggest that the rapid habituation of birds to people wearing masks with a reduced fear response could have some fitness advantage, allowing them to adapt rapidly to the new environmental conditions induced by COVID-19.

## Highlights

• Global lockdown during the COVID-19 pandemic provides an opportunity to study how wildlife responds to changes in human activity.
• Previous work showed that birds in China exhibit reduced fear responses to people with face masks, whereas European studies showed no changes in either rural or urban areas.
• Our results from three Asian countries showed that in both rural and urban areas, birds had a shorter FID to people wearing masks during the COVID-19 pandemic, with birds being more accessible in urban than in rural areas.

## Graphical abstract

The results from three Asian countries showed that whether or not people wear masks (a) makes a difference in the FID of birds in both urban and rural areas, while birds are more accessible in urban than in rural areas (b).

**Figure:**
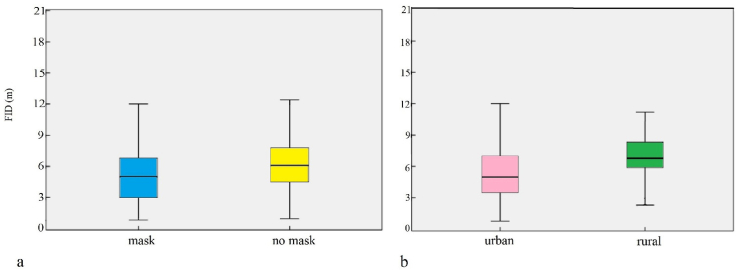

## 1 Introduction

Humans have long played the role of predator [1]. Because the presence of humans can instil fear in wildlife, some animal species or individuals choose to stay away from cities to avoid human disturbance [2]. In spite of this, some species have managed to overcome challenges and thrive in urban habitats [[3], [4], [5]]. Urban ecology research has increasingly focused on figuring out why some species can adapt to urbanization while others cannot [6]. Adaptation to urban environments often implies some phenotypic differentiation from non-urban relatives [3,4,7]. Numerous studies have demonstrated that some animals have even altered certain aspects of their behaviour and life history in response to various disturbances caused by urban human activities, including foraging behaviour, nestling feeding behaviour, activity time patterns, and anti-predatory behaviour [[8], [9], [10], [11]]. These changes in animals can indeed facilitate their adaptation to the rapid environmental changes caused by human activities [7,12]. For instance, animals in urban habitats are frequently contact with people and are disturbed by a vareity of human activities [5], but in reality, humans rarely pose a real threat to urban wildlife, such as birds [13]. Additionally, artificial provisioning increases the amount of food available to wild animals in cities [14,15]. Therefore, repeated exposure to people may serve as a beneficial stimulus for wildlife in urban ares. Reduce antipredator responses to humans in the wild are caused by urban habitats and feeders [16,17]. Numerous studies have also revealed that urban populations have a shorter flight initiation distance (FID) when faced with human approach than rural populations [ [[18], [19], [20], [21]], but see Ref. [22]].

It has been suggested that the key to the successful settlement of a species in a changing urban environment is the ability of the species to modify its behaviour in response to that habitat [23]. This is due to that flexible behaviour can provide organisms with an advantage in responding to predictable and unpredictable environmental change factors [6,24]. However, the mechanisms by which animals adapt to rapidly changing natural environments are still not fully understood. The global pandemic (COVID-19) is a serious threat to human life and health [25]; therefore, governments have implemented a series of policies to effectively stop the spread of the new coronavirus, such as imposing widespread lockdowns, promoting social distancing, and requiring people to wear masks in public places. These policies have markedly altered daily human behaviour and activity patterns [[26], [27], [28]] and they have also provided an unprecedented opportunity to study how large-scale changes in human activities affect animal behaviour [21,[29], [30], [31], [32], [33], [34], [35]]. The Nubian ibex (*Capra nubiana*) in Ein-Avdat significantly altered its behaviour, when the number of visitors to Ein-Avdat was dramatically reduced in 2020 due to the COVID-19 restrictions [35]. Tree sparrows (*Passer montanus*) had a noticeably shorter FID after continuously exposed to mask-wearing people for six months, according to research by Jiang et al. in China [30]. However, in several European countries, Mikula et al. demonstrated that there was no difference between the FID of birds to masked and non-masked humans [21]. It is evident that the effect of this new element in the environment, specifically the wearing of masks, in triggering the escape response of birds is still controversial. Therefore, it is necessary to repeat these studies in more countries and regions.

COVID-19-causing infections were detected in Pakistan and Bangladesh in February–March 2020 [36]. On December 9, 2021, the city of Xi'an, Shaanxi, China, again found COVID-19 patients, and the city was declared closed again on December 23, 2021. To effectively block the spread of the new coronavirus, China, Pakistan and Bangladesh implemented strict epidemic prevention measures. Both governments required everyone to wear a mask in public places [25]. The governments of Pakistan and Bangladesh also imposed varied degrees of fines punishments for infractions [25]. Masks were worn during the lockdown and even after it was lifted. Birds in both countries have been in almost continuously contact with mask users for more than one year as of January 2022. Since Pakistan and Bangladesh are similar to China in terms of service-resistance measures, based on a previous study by Jiang et al. on bird FIDs [30], we hypothesized that birds show less anti-predatory response (e.g., shorter FID) to people wearing masks and are more adapted to them.

## 2 Materials and methods

### 2.1 Study area

**Fig. 1:**
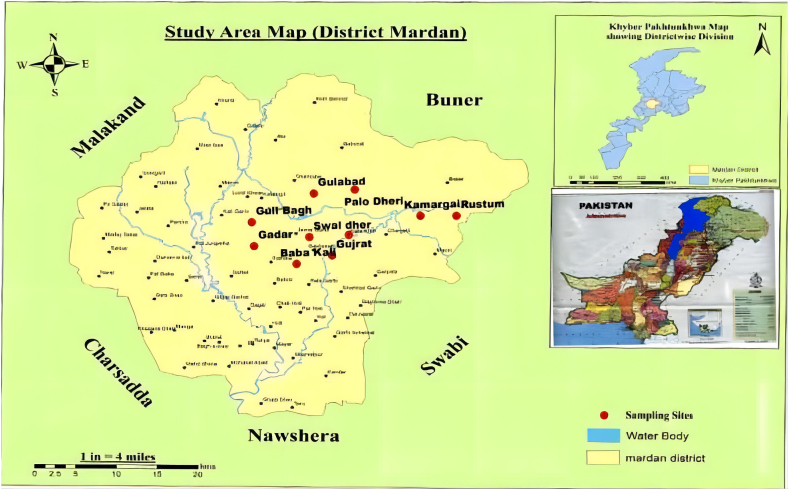
Study area of Mardan, Pakistan.

Data were collected in three Asian countries, Pakistan, Bangladesh and China, and all field experiments were conducted during the non-breeding season, from October 2021 to February 2022. In Pakistan, field data were collected from urban and rural areas in the district of Mardan, in the Pakistani Province of Khyber Pakhtunkhwa (Fig. 1). These two zones were classified as ‘low COVID-19 risk’ zones in this study, and masks were not required in outdoor environments where social distancing of sufficient magnitude was feasible. The entire district covers an area of 632 km^2^. According to the 2017 census, the total population of people residing in Mardan is approximately 2.4 million.

The urban region referred to as Mardan city is located at 34°11′ N, 72°2′ E and consists of well-developed and paved towns, markets, shops, urban parks, and some agricultural fields. Some locations of urban areas are characterised by the presence of human-produced foods on roadside swathes that could also interfere with bird FIDs. The rural area is located outside the city and largely refers to farmed plains located 20 km from Mardan city (34°18′ N, 72°9′ E). In comparison to the urban region, the area has a modest number of settlements, with mainly local farmers. The mosaic of agricultural farmlands and dispersed grasslands with surrounding treed areas are the major focus of the area. The area is less urbanised and populated, with more natural greenery [37].

**Fig. 2:**
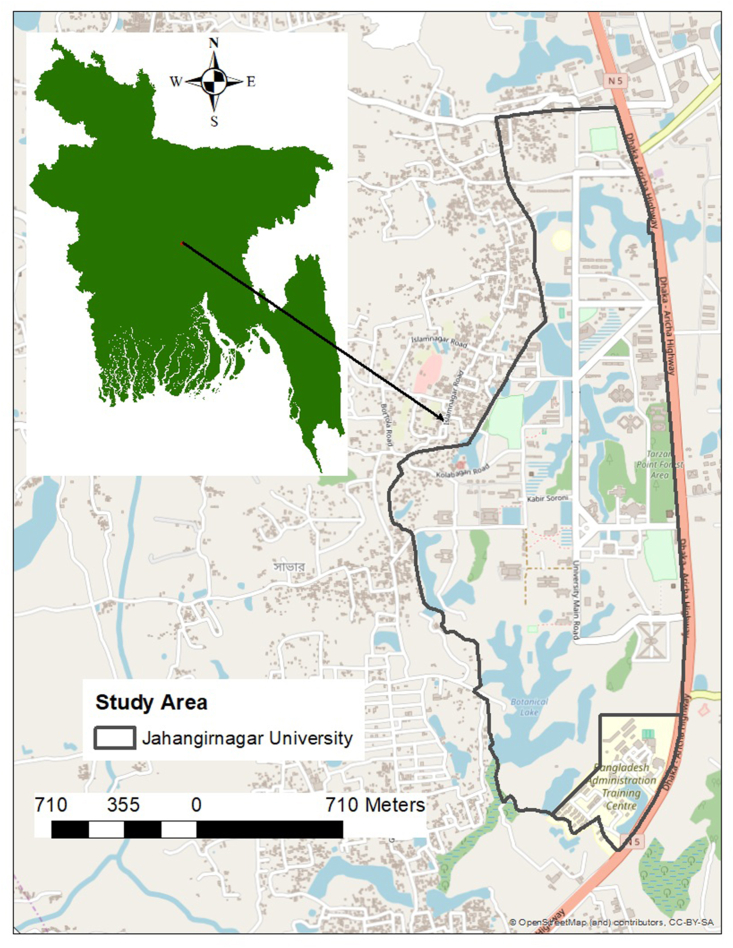
Study area of Jahangirnagar University, Bangladesh.

In Bangladesh, birds were approached in the campus of Jahangirnagar University (23°52′ N, 90°16′ E). The study area was located 32 km north of Dhaka, Bangladesh, and covers an area of approximately 280 ha (Fig. 2). It was completely closed from March 2020 to October 2021. No student was allowed to enter the campus; only teachers and staff who lived inside the campus were allowed, and they more strictly wore face masks more commonly compared to that in other areas of the country. Data of this study site were collected from December 2021 to January 2022.

Xi'an is located in the Guanzhong Basin, between the northern foot of the Qinling Mountains and Weihe River (33°39′ to 34°45′ N, 107°40′ to 109°49′ E) and is an important city in north-western China. Xi'an belongs to the warm temperate sub-humid continental monsoon climate, with a vegetation coverage rate of 29 % [38]. Field data collection in Xi'an was conducted in February 2022.

### 2.2 Field data collection

On a clear day with no wind, an investigator dressed in black walked at a normal walking speed straight towards the target bird moving on the ground. The distance between the target bird and the investigator when it started to take off was recorded as the FID, and the distance was measured in steps and converted to metres. Typically, only a single bird or one from a single species group was measured; birds in mixed flocks were not included. FID data were collected using standardised procedures as detailed by Blumstein [39], and all observers spent at least two months prior to the FID measurement to become familiar with the standardised methods. The process of each observation was taken no longer than three to 5 min. Duplicate sampling of individuals was avoided, and the distance between two collection sites for the same species was more than 50 m.

### 2.3 Statistical analysis

All data analyses were performed with IBM SPSS 22.0 (IBM Corp., Armonk, NY, USA). A generalized linear mixed model with a binomial error distribution was fitted to compare FIDs. To compare FIDs among the three study areas, the FID was used as the response variable, whereas face mask presence and habitat type were used as predictors. When comparing the FIDs of birds within Pakistan, FID was the dependent variable, and habitat type and mask type were independent variables. To test the FID of birds in each habitat type of Pakistan or in Bangladesh and China, FID was used as the response variable, and face mask presence was used as a predictor. All tests were two-tailed, with a significance level determined as *P* < 0.05. The data are presented as the mean ± standard deviation.

## 3 Results

**Fig. 3:**
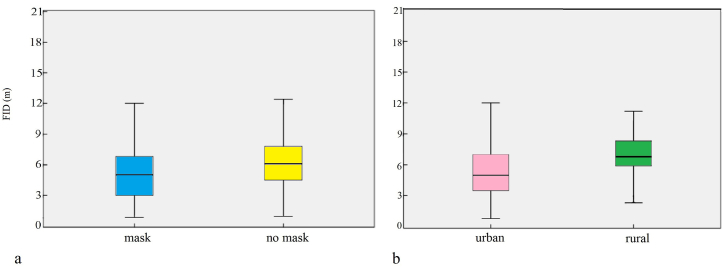
Main fixed effects on flight initiation distances of birds in three countries (a refers to mask type, b refers to habitat type, with error bars showing the standard error).

Excluding species with a sample size fewer than 20, a total of 1822 valid FID datapoints were collected for 13 species in three countries in this study (Table 1). The FIDs collected from the three countries were pooled and analysed to determine the effect of mask wearing and habitat type (urban or rural) on the FID. Results showed that there was a significant difference in FIDs with or without masks (FID with mask: 5.11 ± 2.55 m, FID without mask: 6.51 ± 3.11 m, *P* < 0.001; Fig. 3a). In addition, FIDs differed significantly by habitat type (rural FID: 7.21 ± 2.27 m; urban FID: 5.48 ± 3.00 m; *P* < 0.001; Fig. 3b). Pakistan contains both rural and urban habitat types, whereas the data for China and Bangladesh were from urban areas only. There were differences in FIDs between habitat types, and hence, the FIDs for birds at the three sites were further analysed separately.

**Table 1:** Sample sizes to measure flight initiation distances of birds with and without face masks.

Country	Habitat type	Species	Mask wearing (*n* = )	No mask wearing (*n* = )
Pakistan	Rural area	Collar dove (Streptopelia decaocto)	10	10
		Common babbler (Turdoides caudatus)	10	12
		Common myna (Acridotheres tristis)	50	67
		House crow (Corvus splendens)	55	53
		House sparrow (Passer domesticus)	32	37
		Red-vented bulbul (Pycnonotus cafer)	14	11
		Long-tailed shrike (Lanius schach)	13	13
	Urban area	Bank myna (Acridotheres ginginianus)	54	54
		Common myna (Acridotheres tristis)	65	63
		Feral pigeon (Columba livia)	25	26
		House crow (Corvus splendens)	54	44
		House sparrow (Passer domesticus)	41	66
Bangladesh	Urban area	Asian pied starling (Gracupica contra)	56	58
		Common myna (Acridotheres tristis)	102	173
		House crow (Corvus splendens)	41	31
		House sparrow (Passer domesticus)	35	33
		Jungle babbler (Turdoides striatus)	35	38
		Jungle myna (Acridotheres fuscus)	33	34
		Red-vented bulbul (Pycnonotus cafer)	41	33
China	Urban area	Eurasian tree sparrow (Passer montanus)	96	104

**Fig. 4:**
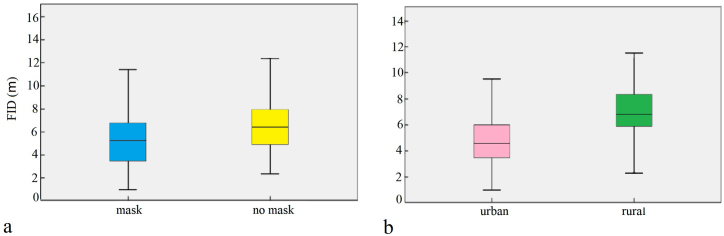
Main fixed effects on flight initiation distances of birds in Pakistan (a refers to mask type, b refers to habitat type, with error bars showing the standard error).

**Fig. 5:**
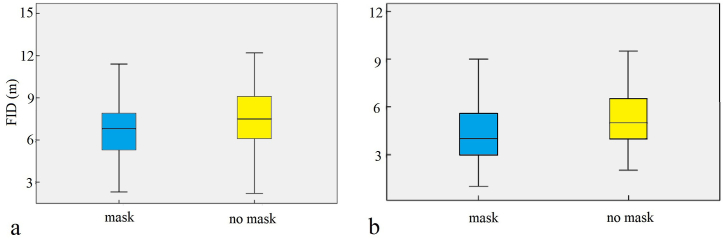
Main fixed effects on flight initiation distances of birds in Pakistan (a refers to mask type of rural area, b refers to mask type of urban area, with error bars showing the standard error).

**Fig. 6:**
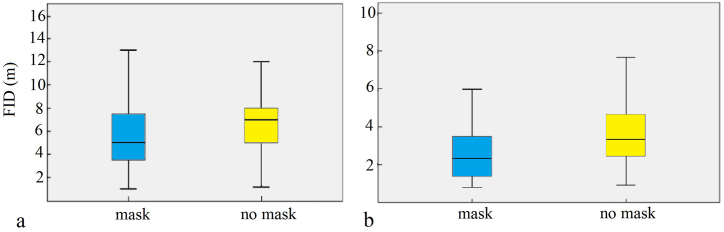
Main fixed effects on flight initiation distances of birds in Bangladesh and China (a refers to mask type of Bangladesh, b refers to mask type of Xi ‘an, China, with error bars showing the standard error).

An analysis of FIDs in Pakistan showed that the FIDs with masks were significantly lower than those without masks (masked FID: 5.30 ± 2.32 m, n = 423; unmasked FID: 6.53 ± 3.06 m, n = 456; *P* < 0.001; Fig. 4a). The urban FID was significantly shorter than the rural FID (rural FID: 7.21 ± 2.27 m, n = 387; urban FID: 4.94 ± 2.77 m, n = 492; *P* < 0.001; Fig. 4b). In Pakistan rural habitats (masked FID: 6.73 ± 2.03 m, n = 184; unmasked FID: 7.64 ± 2.38 m, n = 203; Fig. 5a) and urban habitats (masked FID: 4.20 ± 1.90 m, n = 239; unmasked FID: 5.64 ± 3.24 m, n = 235. Fig. 5b), the FID with masks was significantly lower than that without masks (all *P* < 0.001). For Bangladesh (masked FID: 5.56 ± 2.67 m, n = 343; unmasked FID: 7.22 ± 3.06 m, n = 400; Fig. 6a) and China (masked FID: 2.64 ± 1.49 m, n = 96; unmasked FID: 3.71 ± 1.69 m, n = 104; Fig. 6b), the FID with masks was significantly lower than that without masks (all *P* < 0.001).

## 4 Discussion

This study investigated whether wearing masks as a measure to prevent the spread of the COVID-19–causing virus affects anti-predatory behaviour in birds in central Xi'an, Shaanxi, China, and Jahangirnagar University campus in Bangladesh as well as in the urban and rural areas of Pakistan. The results showed that the FID of birds in both urban and rural areas depends on whether or not people wear masks and birds are more accessible in urban than in rural areas. Although humans often play the role of predators in nature for lengthy periods of history [1,40], capable of triggering fear responses in wildlife and having a marked impact on the behavioural activities of animals [41], they rarely actually pose a real threat to wildlife in cities, such birds. Hence, a reduced fear response to humans may be advantageous for some animals that are regularly exposed to humans [13]. In contrast, birds become more tolerant for humans during repeated exposure to them [16,19 but see 22]. Prior to the outbreak, humans rarely wore masks when travelling. Following the outbreak, masks became a new element in many countries and regions, and this might trigger a neurophobic response (neophobia) in animals [42,43]. However, by October 2021 (when we started collecting data in Pakistan and Bangladesh), birds in two countries, Pakistan and Bangladesh, had been in repeated contact with mask-wearing people for more than one-year. Continuous exposure to harmless stimuli from masked individuals and habituation makes birds more comfortable with masked individuals and less familiar with non-masked individuals [30], as a result, birds had a shorter FID in response to people wearing masks.

Our results were inconsistent with those of Mikula et al. [21], whose results of studies on several bird species in four European countries and in Israel showed “no association between wearing a mask and flight onset distance FID, either in urban or rural areas”. In our study, results from tests in cities in three countries, Pakistan (multiple bird species), Bangladesh (multiple bird species), and China (single bird species), showed shorter FIDs with masks than without masks, and results from tests on multiple bird species in rural areas of Pakistan also showed that people wearing masks were able to approach birds at a closer distance. The possible reason for this is that the five countries where Mikula et al. conducted data collection differed from the three countries we studied in terms of their measures against the COVID-19 pandemic [21]. Anti-epidemic measures were more stringent in the three countries we studied, and masking was still very common even after the policies were lifted for months [25,30]. The contact time with people wearing masks was longer, and the birds were more familiar with masked people and therefore had a shorter FID. In contrast with the research on the Nubian ibex (*Capra nubiana*), which had the exact opposite result, wearing a mask increased the vigilance response [44]. This could be because the study site was a nature reserve, where wildlife is unlikely to come into contact with humans and even less likely to come into contact with people wearing masks. Thus, masks are always a novel stimulus for Nubian ibex, and therefore, they had a longer FID towards masked people. However, in our study, which was conducted during the COVID-19 pandemic, the birds were exposed to mask wearers in both urban and rural settings and were more familiar with them and had a shorter FID.

Our findings support the findings of Jiang et al. [30], but our study includes not only both urban and rural habitats but also a much wider range of species. Our findings further support the pattern of behavioural changes that birds have shorter escape distances from mask wearers than non-mask wearers [30], with convergence across multiple species in regions with similar resistance measures and histories. In addition, our data in Xi'an further showed that even after only approximately two months of exposure to mask wearers, tree sparrows rapidly shifted their fear response to mask wearers and dispalyed a shorter FID. This high degree of behavioural plasticity allows birds to quickly adapt to the rapid environmental changes caused by human activity and thus survive in a changing environment.

Overall, our experiments provide further evidence that birds have a high degree of behavioural plasticity and are able to respond quickly to new stimuli even within a short period of time, which might allow them to adapt rapidly to life with human disturbance, as indicated by Mikula et al. [21]; however, actions taken in response to the COVID-19 epidemic were often short time period. Thus, exploring wildlife responses to environmental changes caused by the COVID-19 epidemic will help us to better understand the mechanisms and ways in which wildlife interact with humans. It would therefore be more meaningful to study animal behaviour in different time dimensions and with different epidemic development patterns. We predict that the FID of birds might differ in cities with recurrent outbreaks, but this needs further validation.

## Ethical standards

The experiments comply with the current laws of Pakistan, Bangladesh and China, where they were performed. Fieldwork was carried out without special permit for this study. Experimental procedures were in agreement with the Animal Research Ethics Committee of Hainan Provincial Education Centre for Ecology and Environment, Hainan Normal University (No. HNECEE-2016-004).

## Data availability statement

Data in this manuscript were provided as supplementary materials (Table S1).

## Funding

This work was supported by 10.13039/100016692Key R & D projects in Ningxia (talent introduction project; 2021BEB04015) and 10.13039/501100012476Fundamental Research Funds for Central Universities, 10.13039/501100012490North Minzu University (2021KYQD05) to JL. WL was supported by the 10.13039/501100001809National Natural Science Foundation of China (No. 32270526) and the 10.13039/501100013142specific research fund of The Innovation Platform for Academicians of Hainan Province. SY was supported by the 10.13039/100017944Hainan Province Postdoctoral Research Project.

## CRediT authorship contribution statement

**Shuang Yang:** Formal analysis, Investigation, Writing – original draft. **Jianping Liu:** Formal analysis, Investigation, Writing – original draft. **Asif Sadam:** Investigation, Resources. **Mominul Islam Nahid:** Investigation, Resources. **Rahmat Ullah Khan:** Investigation, Resources. **Wei Liang:** Conceptualization, Funding acquisition, Supervision, Validation, Writing – review & editing.

## Declaration of competing interest

The authors declare that they have no known competing financial interests or personal relationships that could have appeared to influence the work reported in this paper.
